# Outcomes of Emergency Groin Hernia Repair in the Elderly: A Systematic Review

**DOI:** 10.3389/jaws.2023.11246

**Published:** 2023-06-13

**Authors:** Rodrigo Piltcher-da-Silva, Vivian Laís Sasaki, Luiz Francisco Cravo Bettini, Pedro San Martin Soares, Isabelle Garibaldi Valandro, Leandro Totti Cavazzola

**Affiliations:** ^1^ General Surgery Department, Hospital de Clínicas de Porto Alegre, Universidade Federal do Rio Grande do Sul (UFRGS), Porto Alegre, Brazil; ^2^ Postgraduate Epidemiology Department, Universidade Federal de Pelotas, Pelotas, Brazil

**Keywords:** inguinal hernia, elderly, groin hernia, femoral hernia, emergency surgery

## Abstract

**Introduction:** The number of surgeries for groin hernia (GH) among the elderly follows the increase in life expectancy of the population. The greater number and severity of comorbidities in this group increases the surgical risk, promoting discussion regarding the indication of elective surgery and the benefits of watchful waiting approach (WWA). The aim of the present study was to evaluate the outcomes of emergency hernia surgery among the elderly population.

**Materials and methods:** A systematic review was performed in Pubmed and Scielo databases for the past early 10 years, until July 2022. The subject was groin hernia in the emergency setting focusing the elderly population. The PRISMA statement was followed and the classification of elderly was based on the World Health Organization’s definition.

**Results:** A total of 1,037 results were returned and we ended with nine original articles with emphasis in groin hernia in the emergency among the elderly population. In these subjects, the complications rate ranged between 21.2% and 28.9% and the mortality rate ranged between 1.2% and 6%. Cardiopulmonary disease, high ASA and Charlson’s scales were associated with greater risk of complications and death.

**Conclusion:** Emergency GH surgery in the elderly population carries an increased risk of complications and mortality. GH surgery is safe or, at least, less harmful when done electively. The risk and benefits of WWA and upfront surgery needs to be assessed and exposed to the patients. Our review sugest that elective surgery should be the option over WWA in this patient population.

## Introduction

Groin hernia (GH), referring in this paper for both inguinal and femoral hernias, surgery is one of the most common surgeries worldwide. More than 20 million procedures are performed annually [1–3]. The lifetime occurrence of groin hernias is 27%–43% in men and 3%–6% in women, being more prevalent in elderly patients [4–6]. Nowadays, the increase in life expectancy and the focus on quality of life have brought greater importance to this subject, which has a negative impact on psychological, physical activity and in general wellbeing.

GH incarceration or strangulation has a high incidence among the emergency surgeries, and it is a challenge when present in an elderly and frail patient [2, 4]. The estimated risk of an inguinal hernia incarceration is 4.5% in 2 years, and it is as high as 22% in 3 months for femoral hernias [4]. Strangulation is the progression of hernia incarceration in which there is compromised blood flow to the contents of the hernia sac and ischemic process, is present in 15% of patients and emergency surgery will be mandatory [1, 2]. In the elderly 40% of hernia surgeries are performed due to incarceration, strangulation, or bowel obstruction [7].

Most GH repairs are treated by elective surgery with a mortality of 0.1%, whereas in the emergency it ranges between 1.7% and 7% and with a morbidity in up to 50% of cases. But for elderly and multi comorbid patients, watchful waiting approach (WWA) is recommended by many surgeons, being a risk and benefit assessment situation [1, 7–9]. Thus, the better approach is still a matter of debate, since there is a narrow line between the hazards and advantages of WWA *versus* elective surgery in this specific population [4].

The gold standard treatment for inguinal hernia is a tension-free repair. However, in emergency surgeries, tissue repair has been used for strangulated hernia when there is concomitant bowel resection or field contamination [4]. Mesh is used to reduce the incidence of recurrence [3]; however, due to the mesh related complications and the life expectancy, the advantages of mesh reinforcement are questionable.

The aim of the present study was to evaluate the outcomes of emergency hernia surgery in the elderly population.

## Methods

**FIGURE 1:**
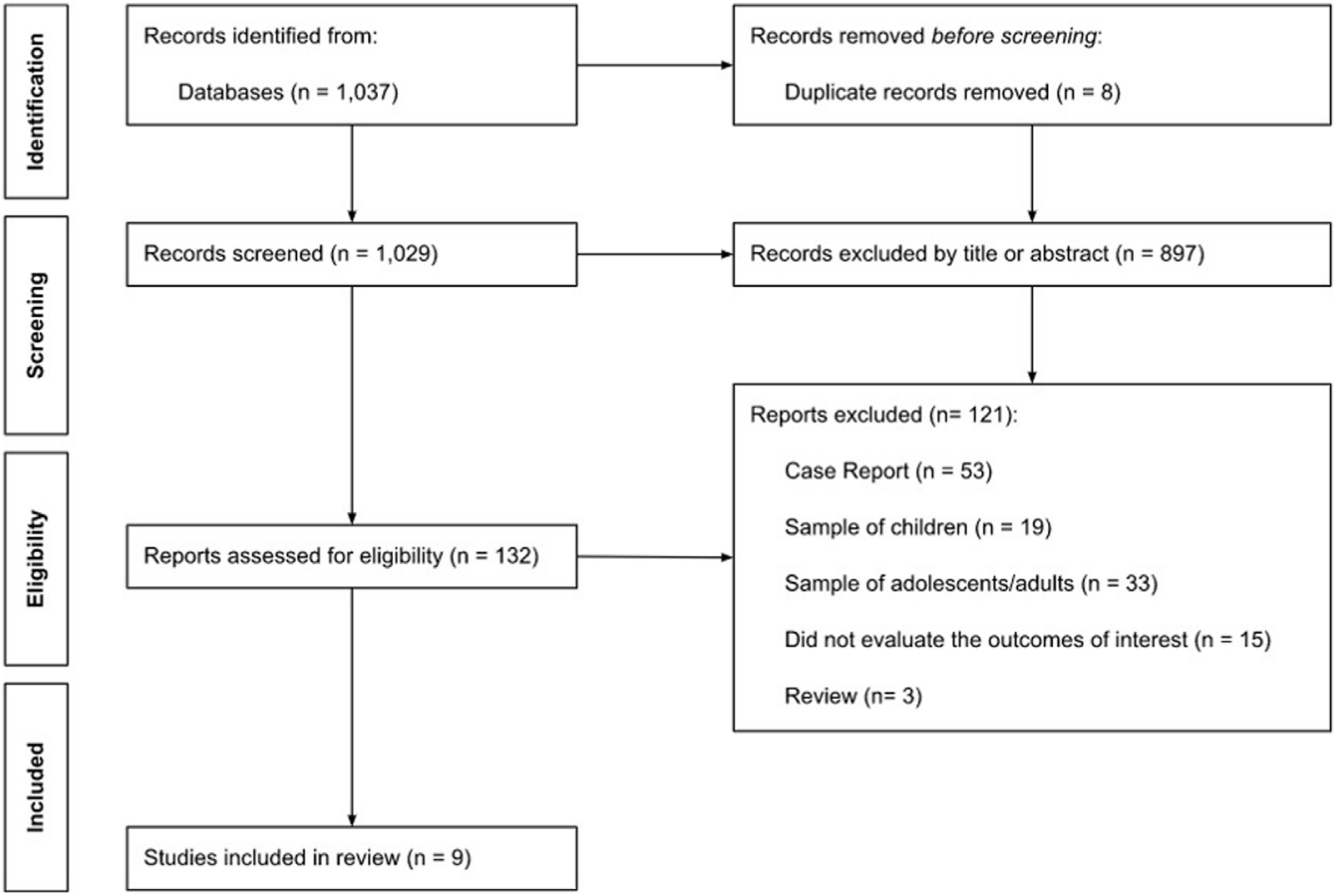
Flow chart.

This systematic review was conducted according to the PRISMA statement [10]. The systematic search was performed in the databases PubMed and Scielo as well as in the reference lists of the eligible articles, published up until 04 July 2022. The search used terms and Boolean operators as follows (“INGUINAL HERNIA” or “GROIN HERNIA” or “FEMORAL HERNIA”) and (“EMERGENCY” or “incarcerated” or “strangulated”; Figure 1). We excluded review articles, systematic reviews, editorials, and commentaries unless they contained original empirical results. Only articles written in English and published between 2012 and 2022 were included in this review.

We included observational studies that assessed the relation between risk factors and outcomes of emergency groin hernia repair in the elderly as well as complications that were most likely to be associated with emergency groin hernia repair in the elderly. Our classification of elderly was based on the World Health Organization’s (WHO) definition, which is defined as 60 years of age or older (World Health Organization, 1986).

All references were imported into the literature management program Rayyan^®^. After reviewing the reference lists of the identified studies and removing duplicates, two review authors performed article selection independently who screened through the titles, abstracts, and full-text citations (RP-d-S and VS). In case of discrepancies, study exclusion was determined after discussion. We did not perform a meta-analysis due to methodological diversity of the included studies and presentation of results.

## Results

### Search Results

The flow of citations through the systematic review process is shown in Figure 1. A total of 1,037 results were returned. After removing eight duplicates, this search retrieved 1,029 unique citations. A total of 897 papers were rejected at title and abstract level. Subsequently, full-text copies of 132 potentially relevant citations were obtained and reviewed. Of these 132 papers, a total of 123 articles were excluded. Therefore, nine unique citations passed the eligibility criteria and were included in the systematic review (Figure 1).

#### Characteristics of the Included Studies

Table 1 describes the characteristics of the included studies. The studies were conducted between 2013 and 2022, with varying sample sizes ranging from 48 to 21,602 patients. Two studies were conducted in the United States, two in Italy, and the remaining studies took place in Saudi Arabia, Hong Kong, Turkey, Israel, and Japan. The majority of the studies focused on men aged 65 years and older.

**TABLE 1:** Characteristics of the included studies.

Author	Year	Country	Study design	Sample size	% Men	Age group	Findings
Bal et al.	2022	United States	Retrospective longitudinal observational	21,602	87.0	≥70 years	Mortality occurred in 16 patients (0.1%) who underwent emergency laparoscopic or open inguinal hernia repair
Ceresoli et al.	2022	Italy	Prospective longitudinal observational	259	57.9	≥65 years	Mortality was observed in seven patients (2.7%) following emergency hernia repair
Akeel	2021	Saudi Arabia	Retrospective longitudinal observational	262	95.0	>60 years	The postoperative mortality rate was 0%
							Patients with higher scores on the American Society of Anesthesiologists (ASA) scale were more likely to experience complications following emergency groin hernia repair
							Female gender was associated with an increased risk of complications after emergency groin hernia repair
Chia et al.	2017	Hong Kong	Retrospective longitudinal observational	190	29.5	≥70 years	Mortality ranged from 3.7% to 6.8% across the three groups and was similar among all groups
							The rate of acute coronary syndrome was 7.3%
							The rate of urinary infections was 3.2%
							The overall complications rate was 28.9%
Işıl et al.	2017	Türkiye	Retrospective longitudinal observational	1,824	89.5	>65 years	The odds ratios for mortality after emergency groin hernia repair were 26.3%
Azari, Perry, and Kirshtein	2015	Israel	Retrospective longitudinal observational	200	67.5	≥60 years	Mortality rates were 0% in patients younger than 59 years, 5.3% in the 60–79 years age group, and 12.1% in patients aged 80 years and older
							The rate of respiratory disease after emergency groin hernia repair was 4.5%
Koizumi et al.	2014	Japan	Retrospective longitudinal observational	93	53.8	≥60 years	Postoperative mortality rate was 0%
							The overall complications rate was 27.9%
Compagna et al.	2013	Italy	Retrospective longitudinal observational	48	29.1	≥75 years	The mortality rate was 6.6%
							The rate of urinary infections was 60.4%
							The rate of respiratory disease after emergency groin hernia repair was 31.3%
Pallati et al.	2013	United States	Retrospective longitudinal observational	2,377	81.4	≥80 years	Mortality is ten times higher in nonagenarians compared to octogenarians in elective inguinal hernia repair (3% vs 0.3%)
							Emergency repair was associated with higher mortality (odds ratio 13.9, 95% confidence interval 5.4–35.5)

#### Risk Factors Associated With the Outcomes of Emergency Groin Hernia Repair in the Elderly

Three studies investigated the risk factors associated with the outcomes after emergency groin hernia repair in the elderly. In a study by Akeel [11], being female was associated with complications after emergency groin hernia repair. In a study by Ceresoli et al. [4], tachycardia, Mental impairment, Charlson ≥6, and laparotomy were positively associated with complication, major complication, and mortality after emergency groin hernia repair. In a study by Işıl et al. [9], end-stage renal disease was associated with complications and mortality after emergency groin hernia repair. Patients who had chronic obstructive pulmonary disease (COPD) were 2.5 times more likely to have complications in study by Ceresoli et al., whereas, in study by Işıl et al., the odds ratios for mortality after emergency groin hernia repair was 26.3. Patients who had higher scores on the American Society of Anesthesiologist (ASA) scale were more likely to have complications after emergency groin hernia repair [4, 9, 11].

#### Complications After Emergency Groin Hernia Repair in the Elderly

Five studies assessed which complications were most likely to be associated with emergency groin hernia repair in the elderly. The overall complications rate ranged between 21.2% and 28.9% [4, 12–14]. In four studies, the respiratory disease rate after emergency groin hernia repair ranged between 3.1% and 31.3% [4, 12, 13, 15]. In three studies, heart complications rate, such as ischemic heart disease [4, 15], acute coronary syndrome [12], and arrhythmia [4], ranged between 1.2% and 10.4%. In addition, urinary infections rate in study by Compagna et al. [15] and Chia et al. [12] was 60.4% and 3.2%, respectively. The occurrence of the other complications studied were inexpressive (<1.0%).

The mortality rate ranged between 1.2% and 6% [4, 8, 13, 15]. In study by Compagna et al. [15], mortality rate was greater in those who were over 75 years. Similar finding was found by Pallati et al. [16].

## Discussion

Elderly patients are at greater risk for groin hernia development than the general population due to abdominal wall loss of strength, comorbidities, and conditions that increase intraabdominal pressure [7, 9]. The incidence of emergency hernia repair is increasing in advanced age patients as life expectancy has increased and surgery is delayed in some cases, which can lead to deadly outcomes [1, 2].

The associated factors for groin hernia incarceration/strangulation are advanced age, obesity, higher ASA score, recurrent hernia, and femoral hernia [1, 4]. Large defects, European Hernia Society classification III (EHA III: >3 cm), are associated with emergency surgery with a 2-fold higher incidence compared to their percentage among elective repairs [1].

Elderly patients are more susceptible to surgical complications and mortality due to comorbidities such as: diabetes, hypoproteinemia, coronary artery disease, cardiac insufficiency, arrhythmias, chronic obstructive pulmonary disease, smoking, wheezing, dyspnea, and impaired mental status [2, 4, 7, 8]. Ceresoli et. al. evaluated 259 patients operated for groin hernia. The mean age was 80 (±8) years and found an overall mortality of 2.8% and it increased to 7.14% for those who needed laparotomy and bowel resection, which is also found in our research. There reports of mortality up to 20% in cases of bowel resection due to ischemic process [17]. Major complications were higher when compared to elective surgery, getting around 5% [4, 18]. A strict postoperative follow-up is necessary since the risk of cardiac complications (myocardial ischemia/infarction), pulmonary system impairment, cerebral and cognitive complications is high [19]. These postoperative situations commonly originated from comorbidities decompensation [7], which reinforce the advantages of multidisciplinary assessment in the perioperative period.

An important concern related to the surgery is the risk of chronic pain of up to 8% which decreases the patient’s quality of life [3, 8]. So, WWA was recommended by the European Hernia Association for asymptomatic and mild symptomatic patients with comorbid conditions if the risk of hernia related emergency is low [20].

However, 70% of patients in WWA will need surgery within 5 years due to complications or worsening of symptoms [4]. Therefore, if there are risk factors for incarceration/strangulation, caution is advised when choosing WWA, since the postoperative complications are higher in emergency treatment [4, 7].

In Wu et. al., a database analysis with 19,683 patients, a significant increase in the mortality odds was found in all age groups when comparing elective and non-elective surgeries. The results are astonishing especially within the 80 and older age group going from 0.19%, in the elective settings to 10.3%, in the emergency settings. Isil et. al. also describes that not only is the mortality higher on geriatric patients but also the period on ICU/hospital stays and the incidence of postoperative morbidity (1% on elective patients vs. 24% on emergency patients).

ASA score and Charlson’s comorbidity index could be used as a complementary tool to predict surgical risks on the emergency hernia repair [4]. In this review, only one article has found an association between Charlson comorbidity index greater or equal to 6 with greater number of surgical complications [4]. This stratification in preoperative evaluation will assess the elderly patients who are candidates for elective hernia repair over WWA, avoiding the additional risk of emergency surgery.

There are many techniques for GH repair. Classically, the Lichtenstein technique was the first option to the repair of incarcerated and strangulated groin hernias with clean or clean-contaminated [4]. The MIS is worldwide accepted as the first approach for most cases of elective hernia surgery, with the advantages of early recovery, good cosmetic results, and better patient related outcomes [2]. Nowadays, MIS has emerged as a viable option for emergency hernia surgery; so, totally extraperitoneal (TEP) and transabdominal pré-peritoneal (TAPP) approaches became a possibility [1, 2]. Because the possibility of abdominal cavity inventory, TAPP is the most common choice for emergencies when MIS is chosen, being of great help especially in cases of inadvertent reduction of hernia content occurred after sedoanalgesia [1, 2].

Depending on process evolution time, the surgical field can present cellulitis, purulent secretion or even fecal or intestinal secretion. For these patients or in those with ischemic bowel and dirty or contaminated field, tissue repair without mesh is encouraged, once the infection rate is up to 38% following bowel resection [2, 4]. Bassini’s or Shouldice’s techniques are some of the surgical techniques used for inguinal hernia repair and MacVay’s technique is the most common surgical procedure for femoral hernia. The age must be evaluated when the surgical technique is chosen. For example, the risk of recurrence appears to be mitigated in multi comorbid elderly due to lower life expectancy. So, mesh may be dispensable, avoiding postoperative complication with mesh infection [2].

An analysis of 13,028 patients who underwent emergency hernia surgery between 2010 and 2019 showed that in 2019, the Lichtenstein technique remains as the most common procedure for incarcerated/strangulated hernias without bowel resection (39.2%) followed by TAPP (37.4%). Nonetheless, tissue repairs were the most common option when bowel resection was necessary [1].

Among the limitations in the included studies are the small sample sizes, which may reduce the statistical power and restrict the possibility of stratified analyses or generate selection bias as well the heterogeneity that contributes to a low quality of evidence.

## Conclusion

With improvement of GH technique, elective repair in elderly patients is acceptable and less harmful over WWA, in the view of the increased morbidity and mortality in an emergency setting. This analysis highlights that clinical complications can provide a worse end result in an incarcerated hernia repair, but can be managed pre-operatively in an elective scenario. Thus, risks and benefits of upfront surgery need to be assessed using measures of number and severity of the comorbidities and discussed with the patient.
